# Risk of malignancy and diagnostic accuracy of fine-needle aspiration biopsy in thyroid nodules with diameters greater than 4 centimeters

**DOI:** 10.20945/2359-3997000000644

**Published:** 2023-06-19

**Authors:** Rafaela N. Barcelos, Cléber P. Camacho, Maria da Conceição de O. C. Mamone, Elza S. Ikejiri, Felipe A. B. Vanderlei, Ji H. Yang, Rosália P. Padovani, Leandro A. L. Martins, Rosa Paula M. Biscolla, Danielle Macellaro, Susan C. Lindsey, Rui M. B. Maciel, João Roberto M. Martins

**Affiliations:** 1 Universidade Federal de São Paulo Escola Paulista de Medicina Departamento de Medicina São Paulo SP Brasil Centro de Doenças da Tireoide e Laboratório de Endocrinologia Molecular e Translacional, Disciplina de Endocrinologia e Metabologia, Departamento de Medicina, Escola Paulista de Medicina, Universidade Federal de São Paulo, São Paulo, SP, Brasil; 2 Universidade Nove de Julho Laboratório de Inovação Molecular e Biotecnologia São Paulo SP Brasil Laboratório de Inovação Molecular e Biotecnologia, Programa de Pós-graduação em Medicina, Universidade Nove de Julho (Uninove), São Paulo, SP, Brasil; 3 Hospital Israelita Albert Einstein Laboratório de Anatomia Clínica e Patológica São Paulo SP Brasil Laboratório de Anatomia Clínica e Patológica, Hospital Israelita Albert Einstein, São Paulo, SP, Brasil

**Keywords:** Thyroid nodule, fine-needle aspiration biopsy, thyroid ultrasound, diagnostic accuracy of fine-needle aspiration biopsy, risk of malignancy

## Abstract

**Objective:**

The risk of malignancy and diagnostic accuracy of fine-needle aspiration biopsy (FNAB) of thyroid nodules (TN) with diameters ≥ 3-4 cm remains controversial. However, some groups have indicated surgical treatment in these patients regardless of the FNAB results. We aimed to evaluate the diagnostic accuracy of the FNAB in systematically resected ≥4 cm TN and if the risk of malignancy is higher in these patients.

**Subjects and methods:**

We retrospectively evaluated 138 patients (142 nodules) with TN with diameters ≥4 cm who underwent thyroidectomy.

**Results:**

The FNAB results were nondiagnostic/unsatisfactory (ND/UNS) in 2.1% of the cases and benign in 51.4%. They indicated atypia of undetermined significance/follicular lesion of undetermined significance (AUS/FLUS) in 23.9% of cases, follicular neoplasia/suspicious for a follicular neoplasm (FN/SFN) in 9.2%, suspicion of malignancy (SUS) in 8.5%, and malignant in 4.9%. The histopathological analysis after thyroidectomy revealed a thyroid cancer rate of 100% in the FNABs classified as malignant, 33.3% in SUS cases, 7.7% in FN/SFN, 17.6% in AUS/FLUS, and 4.1% in benign FNABs. None of the ND/UNS FNABs were malignant. The global malignancy diagnosis was 14.8% (n = 21). However, the rate of false negatives for FNAB was low (4.1%).

**Conclusion:**

We showed that the risk of malignancy in nodules with diameters ≥4 cm was higher compared to the risk of thyroid cancer in TN in general. However, we found a low rate of false-negative cytological results; therefore, our data do not justify the orientation of routine resection for these larger nodules.

## INTRODUCTION

Thyroid nodules (TNs) are common clinical findings, with a prevalence of physical examination of approximately 5% in women and 1% in men, particularly in areas where iodine intake is normal (1, 2). However, the advent of thyroid ultrasound (US) has completely changed the panorama of thyroid nodule diagnosis. When used in routine examinations, this tool identifies thyroid nodules in 37-68% of the population, depending on gender and age (3–5). As a result of this overdiagnosis, the incidence of thyroid cancer has tripled in the last three decades. This increase is almost entirely due to microcarcinomas (<1 cm in diameter), which have an excellent long-term prognosis (6, 7).

The main complementary exam in the investigation of a TN is fine-needle aspiration biopsy (FNAB) (8). This test is indicated depending on the nodules’ size and characteristics as identified using US (1). When performed by an experienced physician, FNAB provides reliable information; however, in some situations, the cytological study may be inconclusive, either because the material is insufficient or due to the lack of morphological diagnosis criteria (1).

In recent years, less accurate FNAB has been described in nodules larger than 3-4 cm. Some researchers even claim that the FNAB, in these cases, is unreliable (9–16) and that the prevalence of malignancy in these larger nodules is higher than in smaller nodules (17–19). This issue is significant because several researchers recommend the surgical removal of nodules with diameters ≥4 cm regardless of cytology results (9,13,17,19,20). However, this issue remains controversial. Other studies have found no difference in the frequency of malignancy and the proportion of FNAB false-negative rates when comparing nodules larger than 4 cm in diameter with those that are smaller (21–30). Thus, this study aimed to evaluate the accuracy of cytology results obtained by FNAB in nodules with diameters ≥4 cm and whether the risk of malignancy is greater in these cases.

## SUBJECTS AND METHODS

### Patients

In this study, we retrospectively evaluated data from 666 thyroid nodules from 500 patients. Of these, we included 142 nodules with diameters greater than or equal to 4 cm (from 138 patients). All patients with a thyroid nodule ≥ 4 cm underwent thyroidectomy. Patients with hyperfunctioning nodules based on scintigraphy results, previous differentiated thyroid carcinoma (DTC), medullary thyroid carcinoma, familial non-medullary thyroid carcinoma (two or more first-degree relatives with DTC), previous thyroidectomy, suspicion of Gardner's syndrome, or incomplete medical records were excluded from the study. All patients attended the Center for Thyroid Diseases (Unifesp) and *Instituto Israelita de Ensino e Pesquisa Albert Einstein* from 2011 to 2014. This study was approved by Unifesp's ethics and research committee (CAAE: 36868514.7.0000.5505).

We recorded information such as age, sex, family history of thyroid disease, exposure to ionizing radiation, compressive symptoms, previous thyroid disease, and laboratory evaluation of thyroid function (TSH) for each patient. Information about the nodules’ size and characteristics was collected using US. All FNABs were guided by US, followed by cytological analysis of the aspirated material according to the Bethesda classification (8). The same professionals performed all US and cytological analyses of the materials obtained from the FNAB. All thyroidectomies were performed by the same team of head and neck surgeons at the Center for Thyroid Diseases.

### Thyroid ultrasound

US was performed using ATL model HDI 3500 equipment (Advanced Technologies Laboratories, USA) and a linear transducer with a frequency of 7.5-10 MHz. US characteristics analyzed included thyroid volume and morphology, number and volume of nodules, content, margin, echogenicity, halo, presence of calcifications, and vascularization on Doppler (31).

### Fine needle aspiration biopsy and cytological analysis

FNAB was performed in single nodules ≥1 cm, regardless of the US characteristics, and in suspicious or dominant nodules if a multinodular goiter is present. For FNAB, 10 mL syringes were used coupled with 20 × 0.55 mm (24 G) needles. The material obtained via FNAB was fixed and stained using the Panoptic technique.

To define the sample as sufficient, we used the criteria suggested by the Guidelines of the Papanicolaou Society, which indicates that the sample must present at least six groups of follicular cells with at least ten cells per group with intact nuclei (32).

The cytological analysis of the aspirate was classified according to the Bethesda System for Reporting Thyroid Cytopathology: non-diagnostic/unsatisfactory (class I), benign (class II), atypia of undetermined meaning/follicular lesion of undetermined meaning (class III), neoplasia follicular/suspicious for follicular neoplasia (class IV), suspicious for malignancy (class V), or malignant (class VI) (8).

### Histopathological

FNAB results were considered false negatives when the nodules had benign cytological and malignant pathological diagnoses. False positives were considered cases whose cytological analyses were class III, class IV, class V or class VI according to the Bethesda System (8) and the benign histopathological analysis.

The same pathological anatomy service performed the histopathological analysis of all cases at the Center for Thyroid Diseases. Given conflicting results (histology versus cytology), the review was performed by more than one professional in the group. We adopt pathological reports according to the 5th edition of the WHO Classification of Endocrine and Neuroendocrine Tumors (33).

### TSH measurement

TSH measurement was performed using a chemiluminescence method (Elecsys TSH, Roche Diagnostics, Mannheim, Germany; reference values: 0.35-5.5 mIU/L with detection limit 0.01 mIU/L).

### Statistical analysis

Quantitative variables were assessed using Student's t-test (or Mann-Whitney test) and Spearman's correlation, as necessary. The proportions were compared using the Q-square test (or Fisher, when necessary). Comparisons between 3 or more groups were made using the Kruskal-Wallis test, with Dunn's post-test. Also, sensitivity, specificity, positive predictive value (PPV), negative predictive value (NPV), and accuracy were calculated according to the methodology used by Galen and Gambino (34). For statistical analysis, we used the SPSS v21 software for Windows, and a significance was set at p < 0.05.

## RESULTS

One hundred thirty-eight patients with 142 thyroid nodules ≥4 cm were operated on consecutively. Of these patients, 13 were men (9.4%), and 125 were women (90.6%). The median age was 46 years (ranging from 19-73 years). No patient reported exposure to ionizing radiation. Forty-four patients had compressive symptoms (31.9%). The median TSH value was 1.16 mIU/L (0.46-5.78 mIU/L). The median follow-up time for the group was eight months (ranging from 2-47 months).

Ultrasonographic characteristics and their ability to diagnose malignancy were obtained for all nodules evaluated and are shown in Table 1. In this analysis, we observed that microcalcification and partial or absent halo could distinguish malignant nodules from benign ones. When these two characteristics were evaluated in an associated way (presence of microcalcifications and missing or partial halo), we also observed a significant difference (p = 0.0001) with a relative risk of 6.8 (95% confidence interval, 2.09-22.11). Sensitivity was 85%, but specificity was 61.6%. The NPV was 95.8%, whereas the PPV was 28.3%.

**Table 1:** Ultrasound characteristics in thyroid nodules and their ability to differentiate between malignant and benign lesions

Ultrasound characteristics	Malignant/benign	Sensitivity (%)	Specificity (%)	PPV (%)	NPV (%)
Microcalcifications (n = 142)	3/0^a^	13	100	100	85.6
Hypoechoic (n = 117)	5/20^b^	23.8	79.2	20	82.6
Partial or missing halo (n = 132)	14/43^c^	82.0	60.0	23.0	96.0
Solid content (n =142)	9/40^d^	45.0	66.0	18.0	88.0
Irregular margins (n = 137)	7/26^e^	33.3	77.6	21.2	86.5

Exact Fisher test

a p = 0.004;

b p = 0.77;

c p = 0.0001;

d p = 0.47;

e p = 0.28.

Abbreviations: PPV: positive predictive value; NPV: negative predictive value.

Partial thyroidectomy was the initial surgical approach in 63 of 138 patients (45.6%); however, three patients (2.2%) underwent a second operation for totalization. In the others (52.2%), the initial surgical approach was total thyroidectomy. Although all patients underwent surgery systematically for nodules greater than or equal to 4.0 cm, in 134 cases (97%), patients already had a formal surgical indication due to the suspected FNAB results, the presence of compressive symptoms, large goiters suspected, cytology in material obtained from FNAB from a nodule other than the most massive, or nodule growth.

Table 2 shows the frequency of malignancy in nodules ≥ 4 cm for each of Bethesda's cytological categories. Overall, most nodules showed a histopathological result of benignity, and the percentage of malignancy in nodules ≥ 4.0 cm was 14.8%.

**Table 2:** Frequency of malignancy in nodule ≥ 4 cm according to FNAB

Cytology	Malignancy rate (%)	Histopathology
Class II	3/73 (4.1)	2FTC, 1FVPTC
Class III	6/34 (17.6)	1PTC, 4FVPTC, 1FTC
Class IV	1/13 (7.7)	1FVPTC
Class V	4/12 (33.3)	2FVPTC, 2FTC
Class VI	7/7 (100)	5PTC, 2FVPTC
Total	21/142 (14.8)	6PTC, 10FVPTC, 5FTC

Abbreviations: FNAB, Fine-needle aspiration biopsy; PTC, classic papillary thyroid carcinoma; FVPTC, infiltrative follicular variant of papillary thyroid carcinoma; FTC, follicular thyroid carcinoma (nomenclature according to the 5th edition of the WHO Classification of Endocrine and Neuroendocrine Tumors, reference 33).

Table 3 shows the clinical and histopathological characteristics of the cases studied. The female-to-male ratio was lower in the comparison of malignant and benign nodules. Also, patients with malignant nodules were younger and had a greater concomitance of Hashimoto's thyroiditis.

**Table 3:** Clinical and histopathological characteristics of the cases studied

	Benign	PTC	FTC	Total	p
	N = 121	N = 16	N = 5	N = 142	
F:M	13.9:1	2.4:1	4:1	9.6:1	0.01^a^
Age (years)					
Average ± SD	46.4 ± 12.4	39.8 ± 8.4	34.0 ± 16.3	45.2 ± 12.6	0.01^b^
Nodule size (cm)					
Average	5.5 ± 1.3	5.4 ± 1.3	6.0 ± 1.7	5.5 ± 1.3	0.81^b^
HT (%)	20/119 (16.8)	6/18 (33.3)	3/5 (60)	29/142 (20.4)	0.021^a^

Abbreviations: PTC, papillary thyroid carcinoma; FTC, follicular carcinoma. F, female; M, male; HT, presence of Hashimoto's thyroiditis in histology; SD, standard deviation;

* Comparison between benign and malignant;

a Exact Fisher test;

b Kruskal-Wallis's test.

The most common histological type was papillary carcinoma (78.3%), and classical and follicular variants were the most frequent (21.7% for both). Only 3 of 21 patients with carcinoma had benign cytological results. FNAB results were considered false positives when classes III-VI had benign histology (66 of 142). Thus, the cytology sensitivity was 87%, with a specificity of 60.3%, PPV of 30.3%, VPN of 95.9%, and accuracy of 64.4%. The false-negative cytology rate in nodules with diameters greater than or equal to 4 cm was 4.1% (p = 0.0005). Table 4 shows the clinical and histopathological characteristics of the 3 cases with false negative results.

**Table 4:** Characteristics of the three patients with false-negative cytology results

	Patient 1	Patient 2	Patient 3
Sex	F	F	F
Age (years)	20	42	24
Single nodule	No	No	Yes
Nodule content	Solid	Mixed*	Mixed*
US size (cm)	9.0	4.1	6.2
Histopathology	FTC	FVPTC	FTC
Size in HP (cm)	8.5	2.0	6.0
Tumor marker	NA	NA	CK19 – fp
			CAL – neg
			Tg – pos
Hashimoto's thyroiditis	No	Yes	Yes
Follow-up (months)	28	39	25

This study evaluated 65 of the 142 nodules (45.8%) as part of multinodular goiter (MNG). The rest of the patients had a single nodule. The percentage of malignancy in the index nodules among patients with MNG was 13.8% (9 of 65), whereas it was 18.2% (14 of 77) in those with unimodular goiter (p = 0.498).

## DISCUSSION

FNAB of thyroid nodules is the main test to distinguish benign nodules from malignant ones (8). It has good sensitivity and specificity and has contributed significantly to the reduction of unnecessary surgeries, especially with the introduction of the US to guide the procedure (35–37). Although FNAB is an excellent diagnostic tool for TNs, some researchers believe the procedure has limitations, as it presents a high rate of false negatives, especially in nodules with diameters ≥ 3-4 cm (38, 39). This subject has been widely discussed in the literature, but no consensus has been reached (40).

In the present study, we showed that the FNAB false-negative rate in TNs with diameters ≥ 4 cm was relatively low (4.1%). Many studies have reported false-negative rates as low as 0-4% (22,25,27,28,30,41). However, for other researchers, the percentages have been as high as 10%-30% (Table 5) (9-16,21). One reason for this disagreement may be how patients are selected for surgery in various studies. Many studies in this area are retrospective, and not all patients with TNs with diameters ≥ 3-4 cm were referred for thyroidectomy. In some studies, only TNs with some characteristics of greater suspicion, such as cytological findings and suspicious US characteristics, nodule growth, and compressive symptoms, were submitted to surgery (14,16,22,24). This could justify the increase in the percentage of false negatives. Other factors potentially involved in these disagreements include the experience of the cytopathologist (42), sampling errors in which the aspirated material did not refer to the index nodule (21, 43), tiny neoplastic foci within a very large nodule (21), and the characteristics of TNs with large cystic components (44). In fact, in the present study, 2 of 3 cases of carcinoma that had benign FNAB results had a cystic component at the US. A similar finding was described by Meko and Norton, who found a 17% false-negative rate in nodules ≥3 cm, which increased to about 30% when the TNs were mixed (31).

**Table 5:** Published literature evaluating false-negative rates in large thyroid nodules with histology

Author	Nodules with benign FNAB (n)	Nodule size (cm)	False negatives rate (%)
Megwalu (30)	62	≥4	0.0
Porterfield and cols. (27)	145	≥3	0.7
Raj and cols. (28)	118	≥4	0.8
Rosario and cols. (23)	84	≥4	3.6
Nam and cols. (41)	632	≥3	3.6
Kuru and cols. (24)	98	≥4	4.1
Cavallo and cols. (25)	37	≥4	5.4
Shrestha and cols. (29)	98	≥4	7.1
Pinchot and cols. (19)	52	≥4	7.7
Al Dawish and cols. (22)	60	≥4	8.3
Bozbiyik and cols. (43)	82	≥4	9.3
Wharry and cols. (9)	125	≥4	10.4
Mehanna and cols. (10)	120	≥3	10.9
Giles and cols. (11)	146	≥4	11.0
Kim and cols. (12)	67	≥4	11.9
McCoy and cols. (13)	71	≥4	12.7
Koo and cols. (14)	14	≥4	14.3
Albuja-Cruz and cols. (21)	113	≥4	15.0
Carrilo and cols. (15)	35	≥4	20.0
Aydogan and cols. (16)	91	≥3	30.0
Present study	78	≥4	3.8

Another controversial aspect of the literature is whether TN size correlates with the malignancy rate. Several studies (9,12,19,21,23–26) have shown that nodules ≥ 3-4 cm in diameter have a higher malignancy rate (12.7%-58.6%) compared to the average percentage of nodule malignancy in general (5%-10%) (1, 2). Some authors, inclusive, have indicated lobectomies for all TNs with diameters ≥ 3-4 cm, regardless of FNAB results (9,13,17,19,20). Here, we showed that the overall malignancy rate was 14.8%, which is similar to the average described in the studies mentioned above (Table 6). However, Nam and cols. (41) found a low malignancy rate (3.6%) in a large series of nodules with diameters ≥3 cm. Similar data have been shown by Sutton and cols. In that study, increasing nodule size > 4 cm and high-risk ultrasound features were not associated with risk of malignancy. Also, authors found that patient age ≥55 years old was independently associated with significantly lower risk of malignancy (45). In addition, Jinih and cols. recently assessed the malignancy rate in thyroid nodules stratified by size (1.0-1.9; 2.0-2.9; 3.0-3.9; 4.0-4.9, and > 5.0 cm). Although the malignancy rate was relatively high (17.6-21.9), it did not differ among the stratification ranges by size (40). On the other hand, Cipriani and cols. showed that, in all nodules ≥ 3 cm (13.1%) and ≥ 4 cm (20.9%), the risk of malignancy was lower than those < 3 cm (19.6%) and < 4 cm (19.9%) (46).

**Table 6:** Published literature evaluating malignancy rate in large thyroid nodules with histology

Author	Nodules with benign FNAB (n)	Nodule size (cm)	Malignancy rate (%)
Nam and cols. (41)	632	≥3	3.6
Raj and cols. (28)	204	≥4	7.8
Megwalu and cols. (30)	90	≥4	10.0
Al Dawish and cols. (22)	79	≥4	12.7
Mehanna and cols. (10)	228	≥3	14.0
Shrestha and cols. (29)	127	≥4	14.2
Aydogan and cols. (16)	130	≥3	14.6
Bozbiyik and cols. (43)	127	≥4	17.3
McCoy and cols. (13)	223	≥4	19.3
Pinchot and cols. (19)	97	≥4	21.6
Wharry and cols. (9)	290	≥4	22.4
Rosario and cols. (23)	151	≥4	22.5
Kuru and cols. (24)	148	≥4	24.0
Cavallo and cols. (25)	92	≥4	25.0
Magister and cols. (26)	63	≥3	33.3
Albuja-Cruz and cols. (21)	212	≥4	35.0
Kim and cols. (12)	263	≥4	58.6
Present study	142	≥4	16.2

Some authors have suggested that the nodules’ US characteristics are more important than their size and are decisive in defining the risk of malignancy (27,47,48). Thus, in a large series of TNs with diameters ≥ 3 cm, Yoon and cols. (47) showed that the rate of false negatives in benign FNAB was low (1.8%). However, US characteristics suggestive of malignancy were more prevalent in FNABs that were malignant or suspected of malignancy than in cytology considered benign (70.3% *vs.* 1.2%). More recently, Hong and cols. also showed that, in a series of 2000 FNABs, the risk of malignancy did not increase as the nodules increased in size. However, patients with TNs with diameters ≥ 3.0 cm had a higher malignancy rate than those with TNs with diameters < 3.0 cm when US characteristics were of intermediate or low risk (18). In the present study, we showed that two US characteristics suggestive of malignancy (microcalcifications and partial or absent halo) could differentiate between malignant and benign TNs with high sensitivity (85%) and negative predictive value (95.8%). This demonstrates the importance of US findings in deciding whether or not to conduct surgery, even if the FNAB is benign.

The prevalence of each Bethesda diagnostic category varies widely in the literature. Class I, II, III, IV, V and VI findings represent 2%-20%, 57%-70%, 3%-8.2%, 2%-25%, 1%-6%, and 3%-14.4%, respectively (8,16,49). The prevalence of each diagnostic category in the present study was 2.1%, 51.4%, 23.9, 9.2, 8.4, and 4.9 for classes I-VI, respectively. In the present series, the prevalence of FNAB in Bethesda category III was higher than that previously described for nodules of all sizes; however, they were similar to those described in studies that evaluated nodules with diameters ≥ 3-4 cm (9,10,19,50). The risk of malignancy was 0%, 4.1%, 17.6%, 7.7%, 33.3%, and 100% for classes I, II, III, IV, V, and VI, respectively. Such findings are similar to those described in the literature, except for non-diagnosed FNABs, which, in several studies, show malignancy rates ranging from 5.3%-54% (8,14,16,19,21, 23,24,28,47,48,51). Perhaps our study's small number of nodules with non-diagnostic cytology explains this disagreement, given that for classes III-VI, positive, sensitivity (87%), specificity (60.3%), PPV (30.3%), NPV (95.9%), and accuracy of cytology (64.4%) were comparable to previous studies (14,29,47).

The histological type of thyroid carcinoma most commonly found in TNs is papillary (85%), followed by follicular carcinoma (12%) and, more rarely, poorly differentiated thyroid carcinomas (<3%) (1). In our study of nodules with diameters ≥ 4 cm, we found a lower percentage of papillary carcinoma (78.3%) than that described for thyroid carcinomas in general. In contrast, the rate of follicular carcinomas was relatively higher (3% of 21%, 14.3%), which is in line with previous studies (11,18,21). Hashimoto's thyroiditis was found more frequently in patients with thyroid carcinomas than in patients with benign pathology. This phenomenon is well described in the literature (52), and hypotheses such as the greater antigenicity of tumor thyroglobulin may justify this finding (53).

The strength of the present study was that the US, FNAB, and surgery were performed by the same group of qualified professionals dedicated to evaluating, diagnosing, and monitoring differentiated thyroid carcinomas. In addition, all patients with TNs with diameters greater than 4 cm underwent thyroidectomy. The weakness of the present study is that it is retrospective and does not evaluate false-negative rates for TNs with diameters less than 4 cm, making it impossible to know whether this rate would be lower in this subgroup of patients.

Finally, in the present study, we show that, although the malignancy rate in TNs with diameters ≥ 4 cm in diameter is high, the rate of benign FNAB false negatives in these same nodules is not high and does not justify the lobectomy or thyroidectomy procedure under such conditions. Further prospective studies with a higher number of cases are necessary to clarify this issue.
